# Kandinsky: Enabling neighbourhood analysis of spatial omics data for functional insights on cell ecosystems

**DOI:** 10.70401/EXO.2025.0017

**Published:** 2026-07-31

**Authors:** Pietro Andrei, Mariachiara Grieco, Amelia Acha-Sagredo, Tommaso Becchi, Pawan Dhami, Kathy Fung, Manuel Rodriguez-Justo, Matteo Cereda, Francesca D Ciccarelli

**Affiliations:** 1 Centre for Cancer Evolution, Barts Cancer Institute, Queen Mary University of London, London EC1M 6AU, UK; 2 Cancer Systems Biology Laboratory, The Francis Crick Institute, London NW1 1AT, UK; 3 Department of Oncology and Hemato-Oncology, University of Milan, Milan 20122, Italy; 4 Italian Institute for Genomic Medicine, c/o IRCCS, Candiolo 10060, Italy; 5 IFOM ETS-The AIRC Institute of Molecular Oncology, Milan 20139, Italy; 6 Genomics Research Platform and Single Cell Laboratory, Guy’s and St Thomas’ NHS Foundation Trust, London SE1 9RT, UK; 7 Department of Research Pathology, UCL Cancer Institute, University College London, London WC1E 6DD, UK

**Keywords:** Spatial biology, cell neighbourhoods, transcriptomics, proteomics, sequencing, imaging, data analysis

## Abstract

**Aims:**

Spatially resolved omics technologies enable investigation of cellular interactions within their local microenvironments (neighbourhoods) directly *in situ*. Although several computational methods have been developed for neighbourhood analysis, significant limitations remain in how neighbourhoods are defined and interrogated. Here, we present Kandinsky, a toolkit that provides a flexible and versatile framework for defining and analysing cell neighbourhoods.

**Methods:**

We developed Kandinsky to improve flexibility in neighbourhood analysis and maximise compatibility with a wide range of spatial omics data. We therefore implemented multiple approaches for identifying cell- or spot-based neighbourhoods that serve as input for four analytical modules: differential gene or protein expression analysis, neighbourhood clustering, co-localisation/dispersion, and spatial hot and cold areas. In addition to its core functionality, Kandinsky enables the execution of external tools within the same analytical framework.

**Results:**

We applied Kandinsky to real and simulated spatial datasets to benchmark its performance against existing methods and demonstrate its ability to uncover biologically meaningful spatial interactions. Kandinsky achieved competitive performance in terms of accuracy, memory usage, and runtime. In real datasets, it suggested transcriptional changes associated with acinar-to-beta cell reprogramming in the healthy pancreas; recapitulated stromal, immune, and tumour-associated clusters in pancreatic cancer; revealed the spatial co-localisation of myoepithelial cells with specific breast cancer subpopulations; and confirmed the association between regions of high *CD74* expression and immune cell infiltration.

**Conclusion:**

Kandinsky is a flexible and versatile toolkit for neighbourhood analysis that facilitates the exploration and interpretation of complex spatial omics data.

## 1 Introduction

Recent technological developments in high dimensional spatial biology open new opportunities to study cell properties and interactions *in situ* at unprecedented levels of resolution and throughput. Spatial proteomic approaches such as imaging mass cytometry (IMC)^[1]^ and co-detection by indexing (CODEX)^[2]^ (now PhenoCycler^[3]^) were the first to enable quantification of tens of proteins at the micro or nano scale using metal-tagged or fluorescent markers. Imaging-based spatial transcriptomic technologies such as MERSCOPE^[4]^, CosMx^[5]^, or Xenium^[6]^ simultaneously track the spatial location of RNAs from hundreds or thousands of genes.

Sequencing-based approaches such as Slide-Seq^[7]^, Visium^[8]^, and Visium-HD^[9]^ detect the whole-transcriptome within capture areas or spots of variable size returning different levels of spatial resolutions. Most recent platforms such as COMET^[10]^ or G4X^[11]^ even enable quantification of both transcripts and proteins from the same cells.

In parallel with technological advances, several analytical tools have been adapted from single-cell analysis or developed *de novo* for spatial data to perform cell segmentation^[12,13]^, deconvolution^[14,15]^, and phenotyping^[16–18]^, as well as inference of cell-cell communications^[19–21]^.

One of the main advances introduced by spatial approaches is the opportunity to investigate the interactions within the local neighbourhood constituted by their proximal cell environment. Neighbourhood analysis has been used to evaluate cell co-localisation or dispersion within the tissue^[22–28]^, map gene co-expression patterns in space^[29,30]^, and identify local cell clusters^[23–26,31,32]^.

An additional aspect that is currently only partially explored is how the local neighbourhood affects the cellular transcriptional profile. Some approaches enable comparison of gene expression between cells of one type grouped according to the presence or absence of a single other type^[33,34]^, whereas others restrict these comparisons to gene expression programmes inferred *a priori* from the data^[22]^. However, current methods provide limited flexibility in defining the cells of interest, their neighbouring cells, and the transcriptional programmes to be compared, thereby restricting the scope of downstream analyses.

To overcome these limitations, we developed Kandinsky, an R package that can work with multiple types of spatial data and offers a variety of approaches to define cell or spot neighbourhoods (c/s-NB). In addition, Kandinsky implements four built-in modules to perform differential gene or protein expression analysis, unsupervised clustering, spatial co-localisation or dispersion, and detection of patterns of high or low gene expression within the tissue (hot/cold areas). In addition, it offers the possibility to run external tools through dedicated wrapper functions, thus enabling a wide range of analyses.

## 2 Methods

### 2.1 Kandinsky data structure and initialization

Kandinsky implements R object-oriented programming defined by an S4 class with 11 components. These components store information regarding c/s-NBs’ identity, cell/spot polygons, c/s-NB definition, c/s-NBs composition, fixed spot distance (spot data), transcript and field of view (FOV) coordinates (cell data), texture features of Haematoxylin & Eosin (H&E) staining, and tissue slide images.

**Figure 1:**
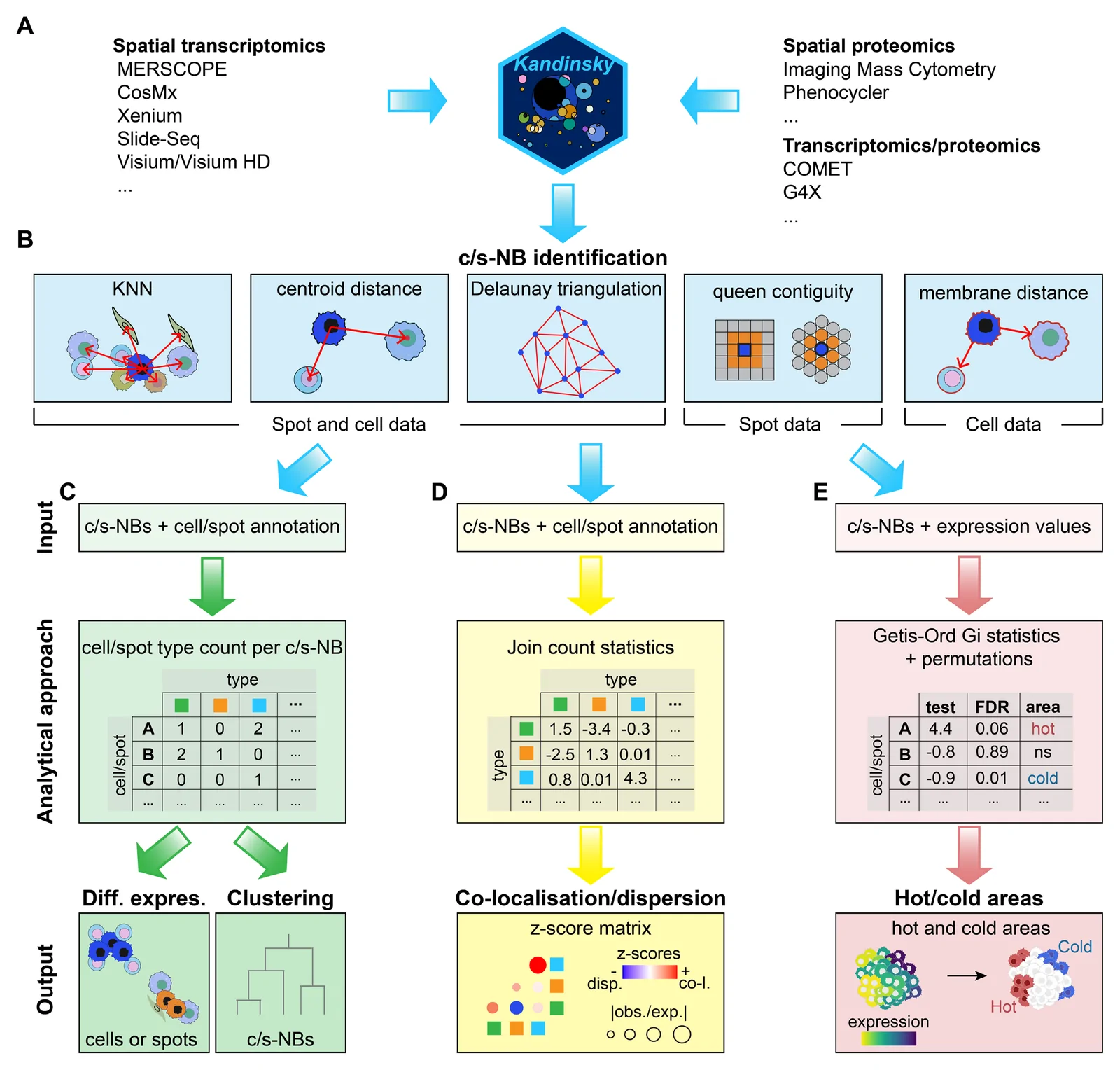
Overview of Kandinsky. (A) Examples of technologies delivering spatially resolved data that can be used as an input for Kandinsky; (B) Representation of the approaches implemented in Kandinsky to identify c/s-NBs based on spatial relationships between cells or spots. KNN, centroid distance, and Delaunay triangulation can be applied to any data type. Queen contiguity and membrane distance can be used only with spot or cell data, respectively; (C) Cells, spots, or c/s-NBs are annotated, and user-defined criteria are applied to a matrix reporting the number of neighbouring cell or spot types surrounding each cell or spot within the tissue. Once defined, groups of cells or spots can be compared in terms of expression levels, or c/s-NBs can be clustered with an unsupervised approach; (D) Cell/spot co-localisation and dispersion. c/s-NBs are used to estimate the tendency of cell or spot types to occupy proximal or distant positions based on z-scores from the multi-class join count test; (E) Hot and cold expression areas. Getis-Ord Gi statistics and permutations are used to identify areas of significantly high and low expression values of a gene, protein, or signature of interest across cells or spots within each c/s-NB. c/s-NB: cell/spot neighbourhood; KNN: K-nearest neighbour; FDR: false discovery rate.

Class construction is implemented in two steps. First, Kandinsky exploits a set of platform-specific *prepare_seurat* functions which read raw data files from different spatial technologies to initialize a Seurat object. Second, the *kandinsky_init* function creates the object that is stored within the object containing polygon, neighbour and image data associated with the spatial dataset. If a Seurat object has already been created prior to Kandinsky, *kandinsky_init* can still be called if all the required input files are provided. The final output is an updated version of the input Seurat object with the new Kandinsky object stored as a ‘tools’ slot. (Figure 1A).

### 2.2 c/s-NB identification

The *kandinsky_init* function uses cell or spot coordinates to derive cell or spot polygons and identify c/s-NBs relying on R packages sf v1.1.0^[35]^, spdep v1.4.2^[36]^, and tripack v1.3.9.4^[37]^ based on five neighbouring methods (Figure 1B):

(1) k-nearest neighbours (KNN) (based on spdep *knearneigh* function), whereby neighbouring cells are selected as the top user-defined k nearest cells;
(2) Centroid distance (based on spdep *dnearneigh* function), whereby cells or spots with centroids closer than a user-defined distance are considered within the same c/s-NB;
(3) Delaunay triangulation (based on tripack *tri.mesh* function), whereby c/s-NBs are the edges of a network of triangles whose nodes are cells or spots;
(4) Queen contiguity (spot data only, based on spdep *poly2nb* function), whereby s-NBs are defined as the top user-defined number of layers of surrounding spots;
(5) Membrane distance (cell data only), whereby cells with membranes closer than a user-defined distance are considered within the same c-NB. Membrane distances are measured by expanding single-cell boundaries using the *st_buffer* function of a radius *r* calculated as

(1) $$r=\frac{d}{2}$$

where *d* is the user-defined distance.

Neighbours of each cell are identified by checking the intersection between the expanded single-cell boundaries using the sf *st_intersects* function. When independent samples are included in a dataset, c/s-NBs can be defined separately for each sample by specifying a sample labelling scheme through the parameter ‘sample_key’ available in the *kandinsky_init* function.

For Visium and Visium HD technologies, H&E images are loaded as raster objects using the R package terra v1.9.34^[38]^ and aligned to spot coordinates. The resulting list of polygon, c/s-NB, and image objects are assembled into the class as slots, stored as a tool slot within the previously initialised Seurat object, and used as input for downstream analysis.

### 2.3 Differential gene/protein expression module

Input for this module is c/s-NBs and an external annotation of cell or spot types used by the *nnMat* function to generate a c/s-NB composition matrix reporting the count of cell or spot types neighbouring each cell or spot across all c/s-NBs (Figure 1C). This matrix is then used to group cells or spots through the *nn_query* function and perform differential gene or protein expression analysis via the internal call of the *FindMarkers* function.

### 2.4 c/s-NB clustering module

Input for this module is the same as that of the differential gene/protein expression module (Figure 1C). The *nbCluster* function then performs c/s-NB unsupervised clustering based on composition similarity using the R package mclust v6.1.2^[39]^. Users can specify different configurations for the number of clusters to test. For each configuration, the Bayesian information criterion (BIC) is evaluated, and the cluster configuration with the highest BIC value is chosen as optimal. Groups are stored in the metadata and in the sf data slot of the object. Kandinsky also provides wrapper functions to perform c/s-NB clustering with external tools (SpatialLeiden v0.4.0^[40]^, SpatialTopic v1.3.0^[41]^, multiomics and ecological spatial analysis (MESA) v0.1.2^[32]^, ISCHIA v1.0.0.0^[26]^, hoodscanR v1.8.0^[23]^, Giotto v4.2.3^[24]^, CellCharter v0.3.4^[25]^).

### 2.5 Co-localisation and dispersion module

Input for this analysis is c/s-NBs and an external annotation of cell or spot types used by the *jc_coloc* function, that implements the multi-class join count statistics^[42]^ as defined in the *joincount.multi* function from the *spdep* package (Figure 1D). The function calculates the number of observed and expected neighbours between each cell or spot type pair (join counts, *J*) given the overall neighbour composition and under the null hypothesis of random distribution of cell or spot types in the tissue. Then it evaluates if each join occurs by chance by measuring a z-score *z* as:

(2) $$z=\frac{J_{obs\;}-J_{exp\;}}{\sqrt{\text{Var}\left(J_{exp\;}\right)}}$$

where *J*_*obs*_ and *J*_*exp*_ are the observed and expected number of join counts, respectively.

Positive z-score values indicate spatial co-localisation. Negative z-score values indicate spatial dispersion. Additionally, the observed-to-expected ratio *r* of join counts is calculated as:

(3) $$r=\frac{J_{obs}}{J_{exp}}$$

The numerical values of z-scores *z* and their ratio are stored in a tabular format and visualised as a heatmap. Kandinsky also provides wrapper functions to perform co-localisation and dispersion analysis with external tools (Squidpy v1.6.6^[27]^, hoodscanR v.1.8.0^[23]^, Giotto v4.2.3^[24]^, Cellcharter v0.3.4^[25]^).

### 2.6 Hot/cold expression area module

Input for this analysis is c/s-NBs and gene, protein, or gene signature expression values used by the *hotspot_analysis* function to classify cells or spots based on Getis-Ord Gi statistics calculated for each cell or spot using the *localG_perm* function of the spdep package (Figure 1E). Getis-Ord Gi statistics estimate the tendency of cells or spots within the same c/s-NB to express a gene, protein, or signature at significantly higher or lower values than the other cells or spots given the overall c/s-NB composition^[43]^. For each cell or spot *i*, the Getis-Ord Gi statistics are calculated as:

(4) $$G_{i}=\frac{\sum_{j}w_{ij}x_{j}-\sum_{j}w_{ij}\bar{x}}{s(i)\sqrt{\left[\left((n-1)\sum_{j}w_{ij}^{2}\right)-{\left(\sum_{j}w_{ij}\right)}^{2}\right]/(n-2)}},j\neq i$$

given:

(5) $$s(i)=\sqrt{\frac{\sum_{j}x_{j}^{2}}{\left(n-1\right)}-{\bar{x}}^{2}}$$

where *w*_*ij*_ is the spatial weight between the cell or spot of interest *i* and any other cell or spot *j* within the corresponding c/s-NBs, *x*_*j*_ is the expression value of the variable of interest in any cell or spot *j* within the c/s-NBs, $\bar{x}$ is the mean expression of the variable of interest across all cells or spots, *s* is the standardized difference between observed and expected expression values across neighbours, and *n* is the total number of cells or spots considered.

Statistical significance is assessed by comparing each observed value to the expected one calculated after randomly reshuffling expression values across all cells or spots via a permutations test. An empirical p-value is calculated using the spdep *localG_perm* function for each cell or spot as:

(6) $$p=2*\left(\frac{\sum \left(\left|Gi_{\exp}\right|>\left|Gi_{\text{obs}\;}\right|\right)+1}{N+1}\right)$$

where *Gi*_exp_ and *Gi*_*obs*_ are the expected and observed Getis-Ord Gi statistics, respectively, and *N* is the number of permutations. Empirical *p*-values are corrected for multiple testing using Benjamini-Hochberg. Cells or spots associated with a positive or negative Getis-Ord Gi statistics and a false discovery rate (FDR) lower than a user-defined threshold are classified as hot and cold areas, respectively.

### 2.7 Additional functions

In addition to neighbourhood-based analysis, Kandinsky implements additional functions to facilitate spatial data analysis. These include:

(1) *get_visium_textures* function to extract texture features from H&E images associated with Visium and Visium HD data. The R package GLCMTextures v0.4.2^[44]^ is internally used to calculate grey-level co-occurrence matrix texture metrics across pixels. Pixel metrics are then aggregated at the spot level. The output is a spot-metrics matrix stored as a slot of the Kandinsky object, with each metric summarised as the mean pixel-level value observed for each spot;
(2) *he_mask* function to perform automatic tissue masking from H & E images given a pixel filtering threshold specified via the ‘sd_thresh’ parameter;
(3) *global_univ_spatcor* and *global_biv_spatcor* functions to calculate univariate and bivariate spatial correlation coefficients, respectively, for any list of genes or proteins specified by the user;
(4) *stitch_samples* function to merge independent samples into a regular grid and create a common coordinate system.
(5) *create_cell_masks* function to draw tissue mask polygons based on the cell types of interest. The function first calls the R package dbscan v1.2.4^[45]^ to derive density-based clusters of cells of interest and then uses the R package concaveman v1.2.0^[46]^ to draw concave hull masks around each cluster.
(6) *nb_downsize* function to account only for a user-defined number of randomly selected cells or spots per c/s-NB using the ‘exp_links’ parameter. If the initial c/s-NB network is symmetric (as the one produced by queen contiguity, centroid and membrane distance methods), the function will also enforce network symmetry.

### 2.8 CosMx normal pancreas dataset

CosMx Human Whole Transcriptome dataset and annotation file of a human pancreas sample were downloaded from the NanoString website^[47]^. The *prepare_cosmx_seur*at and *kandinsky_init* functions were used to initialize a Seurat object with CosMx raw data. c-NBs were defined during initialization using the membrane distance method (‘nb. method = M’ and ‘d. max’ = 0). The *nnMat* and *nn_query* functions were used to (1) build the c-NB composition matrix with CosMx cell type annotation as reference and (2) separate acinar cells into peri-islet and non peri-islet cells based on the presence or absence of islet cells within the same c-NB. Expression profiles of the two acinar cell groups were compared via differential gene expression analysis using the Seurat^[48]^ *FindMarkers* function directly called via the *nn_query* function. Differentially expressed genes were defined as fold change > 1.1 or < 0.9 and FDR < 0.01 (Bonferroni correction). Genes expressed in < 20% of peri-islet acinar cells and < 5% of non peri-islet acinar cells were excluded to avoid possible contamination.

CellNeighborEX v1.0.1^[34]^ and NicheDE v0.0.0.9^[33]^ were applied to perform differential expression analysis, each with the recommended settings. For CellNeighborEX, differential gene expression analysis was performed using the *CellNeighborEX.DEanalysis.analyzedata* function. Differentially expressed genes were selected based on a logRatio cutoff of 0.4 and FDR < 0.01 after Benjamini-Hochberg correction. For NicheDE, differential gene expression analysis was performed using the *niche_DE* function with 12 cores and default parameters. Differentially expressed genes were selected using the *get_niche_DE_genes* function (test. level = ‘I’, positive = TRUE/FALSE, and alpha = 0.01). Elapsed runtime and memory peak were measured across ten iterations using the R package peakRAM^[49]^ v1.0.3 for Kandinsky and NicheDE or time and memory-profiler python modules for CellNeighborEX.

### 2.9 MERFISH mouse hypothalamus dataset

Five mouse hypothalamic tissue slices profiled with multiplexed error-robust fluorescence in situ hybridization (MERFISH) were downloaded from the SDMBench website^[50]^. For all five slices, ground truth annotations of tissue domains were available and used to benchmark eight spatial clustering approaches: Kandinsky, SpatialLeiden v0.4.0^[40]^, CellCharter v 0.3.4^[25]^, MESA v0.1.2^[32]^, Giotto v4.2.3^[24]^, SpatialTopic v1.3.0^[41]^, hoodscanR v1.8.0^[23]^, and K-means clustering implemented by multiple tools like ISCHIA v1.0.0.0^[26]^ and Seurat v5.5.0^[48]^. For Kandinsky and K-means, a Seurat object was initialized with the *kandinsky_init* function and c-NBs were defined using the KNN method (‘nb. method’ = ’K’ and ‘k’ = 50). For the remaining methods, neighbourhoods were defined using the recommended settings with default parameters.

Tissue domain identification via clustering was performed after aggregating all five MERFISH slices together. Due to the lack of integer count data, dimensionality reduction through single-cell variational inference (SCVI)^[51]^ was not possible for CellCharter and SpatialLeiden, and PCA followed by Harmony integration was performed instead before the domain identification step. Elapsed runtime and memory peak were measured through 10 iterations using the package peakRAM v1.0.3 for R-based software and time and memory-profiler modules for Python-based software.

### 2.10 IMC pancreatic ductal adenocarcinoma (PDAC) dataset

A Seurat object containing all cell measurements and metadata for all IMC regions of interest (ROIs) was downloaded from the Zenodo website^[52]^. Cell centroid coordinates from independent ROIs were shifted and merged into a unique coordinate system using the *stitch_samples* function. Following the original methods^[52]^, a Seurat object was initialized with the *kandinsky_init* function and c-NBs were defined using the KNN method (‘nb. method= K’ and ‘k’ = 20). The *nbCluster* function was then used to group c-NBs across samples, setting the expected number of groups as in the original publication (‘n_clust’ = 10). Enrichment of PDAC cell types or spatial niches in c-NB clusters was tested using Fisher’s test corrected for multiple testing with Benjamini-Hochberg correction.

### 2.11 Simulated spatial dataset

A simulated dataset composed of 2400 samples and four cell types (ct0-ct3) was downloaded from^[53]^. The dataset was built with ct0 showing variable co-localisation preference towards ct1, with ct2 and ct3, which were set as background populations. Samples were generated in the original study using 24 combinations of two parameters: ct0-ct1 co-localisation intensity level *h* (random = 0.25; weak = 0.45; strong = 0.6) and ct0relative abundance *p*_*k*_ (0.05 ≤ *p*_*k*_ ≤ 0.55). For each combination, 100 independent samples were created.

Kandinsky was compared against four other tools: hoodscanR v1.8.0^[23]^, Giotto v4.2.3^[24]^, Squidpy v1.6.6^[27]^, and CellCharter v0.3.4^[25]^ (with *log_fold_change* option set to False or True). Each tool was run using the recommended settings with default parameters. Kandinsky and hoodscanR were executed with a KNN graph (*k* = 10). Giotto was run with a Delaunay graph and with a KNN graph (*k* = 10), while Squidpy and CellCharter shared a single pre-computed Delaunay graph. The neighbour-preference score of each tool for the targeted ct0-ct1 cell pair was used to classify random (*h* = 0.25), weak (*h* = 0.45) and strong (*h* = 0.6) co-localisation. The classification performance was quantified using the area under the receiver operating characteristic curve (AUC-ROC), computed with the *roc* function from the R package pROC v1.19.0.1^[54]^ and the *roc_auc_score* function from the Python module sklearn.metrics, using the ct0-ct1 neighbour-preference score to distinguish between random, weak, or strong co-localisation. Elapsed runtime and memory peak were measured across all 16 conditions, with 10 iterations per condition, using the package peakRAM v1.0.3^[49]^ for R-based software and time and memory-profiler modules for Python-based software.

### 2.12 Xenium human breast cancer dataset

Xenium Prime 5K Human Pan Tissue and Pathways Panel data of a human breast cancer sample were downloaded from the 10X website^[55]^. Raw Xenium data were loaded into R and used to create a Seurat object using the *prepare_xenium_seurat* function. Cells with < 25 transcripts and < 20 unique genes were excluded. A new Seurat object was initialized with the *kandinsky_init* function and c-NBs were defined using the cell centroid distance method (‘nb. method = C’ and ‘d. max’ = 40). For cell clustering, gene expression values were normalised with the *NormalizeData* function with default parameters, and the top 2,000 variable genes were selected using the *FindVariableFeatures* function with default parameters. Based on these genes, the *SketchData* function was applied to select a subset of 75,000 cells representative of the whole dataset. The top 2,000 variable genes were re-selected within the sketched dataset. The *ScaleData* and *RunPCA* functions with default parameters were used to perform dimensionality reduction on the sketched dataset, and the *FindNeighbors* and *FindClusters* functions (‘dims’ = 1:50 and ‘res’ = 1.2) were used to identify cell clusters. Uniform manifold approximation and projection (UMAP) was created using the *RunUMAP* function (‘dims’ = 1:50, ‘return.model’ = T). Single cells excluded from sketching were mapped onto the sketched dimensional reduction results using the *ProjectData* function. Cell type annotation for each cluster was manually curated based on the top differentially expressed genes (fold change (FC) > 1.5 and FDR < 0.05) identified using the *FindAllMarkers* function.

Co-localisation and dispersion of cell types in the tissue were assessed via multi-class join count statistics using the *jc_coloc* function. Differential gene expression analysis between tumour 5 and 6 cells and the rest of the tumour cells was performed using the Seurat *FindMarkers* function for all genes expressed in ≥ 1% of tumour 5 and 6 cells with default parameters. Obtained log2 FC values were used to perform ranked gene set enrichment analysis (GSEA) using the R package fgsea v1.28.0^[56]^.

### 2.13 CosMx human colorectal cancer (CRC) dataset

CosMx raw data and cell annotations of CRC sample CR48 were downloaded from the Zenodo website^[57]^. Raw files were loaded into R and used to create a Seurat object with the *prepare_cosmx_seurat* function. A new Seurat object was initialized with the *kandinsky_init* function and c-NBs were defined using the cell membrane distance method (‘nb. method = ”M”” and ‘d. max’ = 30). Gene expression levels were normalised using the *NormalizeData* function.

Hot and cold areas for *CD74* gene expression were derived using the *hotspot_analysis* function to measure the Getis-Ord Gi statistics and statistical significance with 999 permutations (‘perm’ = 999 and ‘padj. thresh’ = 0.05). Enrichment of tumour associated macrophages (TAMs), CRC, and T cells/Natural Killer (T/NK) cells within *CD74* hot and cold areas was tested using one-tailed Fisher’s exact test. The tumour mask was created using the *create_cell_masks* function with default parameters (‘eps’ = 30, ‘minPts’ = 6, ‘concavity’ = 3, ‘lengthThreshold’ = 0, ‘buffer’ = NULL), considering CRC cells as the cell type of interest.

To assess the effect of T/NK cell proximity on *CD74* expression in CRC cells, the *nnMat and nn_query* functions were run to define the contribution of CRC cell types within each c-NB and group CRC cells based on the presence or absence of T/NK cells within the c-NBs. *CD74* expression between CRC cells proximal to T/NK cells and the rest was compared using the two-tailed Wilcoxon’s rank-sum test.

### 2.14 Visium CRC data generation and analysis

A 5 μm section was cut from the formalin-fixed paraffin-embedded (FFPE) block of CRC sample CR48 and placed within the 6 × 6 mm^2^ fiducial frame of the Visium slide. The slide was then incubated at 42 °C for 3 h, deparaffinized, H&E stained, and imaged using an Olympus VS200 slide scanner. Once imaged, the coverslip was removed, and the slide was decrosslinked. The Visium Human Transcriptome Probe kit (v1, PN-1000363) was used for transcript hybridisation. Hybridised RNA molecules were released after tissue permeabilization and captured within each spot by barcoded oligonucleotides. Captured RNA molecules were used for sequencing library preparation and sequenced using the NextSeq 2,000 with a sequencing depth of ≥ 25,000 reads per tissue covered spot.

Visium FASTQ files were processed using the 10X Genomics spaceranger v2.0 software to derive raw gene expression count data. Visium gene expression data were imported into R using Seurat^[48]^. Spots matching empty regions within the tissue slides annotated using the 10X Genomics Loupe Browser v6.2.0 software and those with < 500 transcripts and 300 unique genes were excluded. Genes expressed in less than 10 Visium spots were also removed. Visium raw counts were normalized using the *NormalizeData* function. A new Seurat object was initialized with the *kandinsky_init* function. s-NBs were defined using the queen contiguity method (‘nb. Method = ”Q””) and the *nb_expand* function to add the second closest spot layer.

Gene expression scores for the seven immune gene signatures (M0-27, CXCL10/TAM-16, CD209/TAM-13, T-8, CD4-17, Cytotoxic-23, NK-33) were calculated and smoothed across spots using the UCell^[58]^ *AddModuleScore_UCell* (‘maxRank = 5,000’) and *SmoothKNN* (‘k’ = 20) functions, respectively. Hot and cold areas for the immune signatures and *CD74* gene were defined using the *hotspot_analysis* function as described above. Enrichment of immune signatures within *CD74* hot and cold areas was tested via one-tailed Fisher’s exact test and corrected for multiple testing with the Benjamini-Hochberg method.

Voyager v1.8.1^[29]^ and SpottedPy v0.1.1^[30]^ were used with the recommended settings to benchmark Kandinsky performance. For Voyager, hot/cold area detection was performed using the *runUnivariate* function (type = ‘localG_perm’). For SpottedPy, hot/cold area detection was performed using the *spottedpy.create_hotspots* function. Elapsed runtime and memory peak were measured across ten iterations using the R package peakRAM v1.0.3 for Kandinsky and Voyager and the time and memory-profiler Python modules for SpottedPy.

## 3 Results

### 3.1 Kandinsky overview

Kandinsky is an R package for neighbourhood analysis of spatial omics data. Compared to available approaches, it shows higher versatility in terms of type and resolution of input data, neighbour definition, and downstream analyses (Table S1).

Starting from cell or spot coordinates derived from any spatial transcriptomic or proteomic platform, Kandinsky implements helper functions to automate their loading and formatting into Seurat objects (Figure 1A, Methods). It then defines cell or spot neighbourhoods (c/s-NBs) according to the spatial relationships between cells or spots, allowing the user to choose among five alternative methods, depending on the biological question: KNN, centroid distance, Delaunay triangulation, queen contiguity, and membrane distance (Figure 1B). KNN, centroid distance, and Delaunay triangulation are applicable to both cell and spot data. Queen contiguity and membrane distance are limited to spot and cell data, respectively. Once defined, c/s-NBs can be annotated and used to (a) group cells, spots, or c/s-NBs based on user-defined criteria for differential gene/protein expression or spatial clustering (Figure 1C), (b) measure cell or spot co-localisation or dispersion in space (Figure 1D) and (c) derive hot and cold areas within the tissue (Figure 1E).

Cells and spots can be grouped according to user-defined criteria applied to a c/s-NB composition matrix, which reports the number of neighbouring cell or spot types surrounding each cell or spot within the tissue (Figure 1C). For example, one may be interested in extracting all epithelial cells surrounded only by other epithelial cells or immune cells proximal to only a certain type of stromal cells. Once defined, distinct groups of cells or spots can be compared in terms of their global transcriptional profiles or expression levels of specific genes, proteins, or signatures of interest. The same c/s-NB composition matrix can be used to cluster c/s-NBs with an unsupervised approach based on the similarity of their cell or spot composition.

Spatial co-localisation and dispersion measure the tendency of cells or spots of a given type to co-localise with or disperse from other cell or spot types (Figure 1D). In this case, c/s-NBs are used to measure co-occurrence or dispersion between pairs of neighbouring cell or spot types using z-scores and associated observed-to-expected ratios derived using the multi-class join count statistics (Methods).

Lastly, hot and cold areas identify regions of the tissue where genes, proteins, or signatures of interest are expressed more or less than expected by chance (Figure 1E). Here, c/s-NBs are used to detect areas of high or low expression levels across neighbouring cells, using the Getis-Ord Gi statistics, and statistical significance is assessed with permutations (Methods).

In addition to these built-in modules, Kandinsky implements functions to facilitate the analysis of spatial data and to use external tools for spatial clustering and co-localisation, which further enhance analytical versatility.

### 3.2 Neighbourhoods trigger transcriptional changes in healthy pancreas

**Figure 2:**
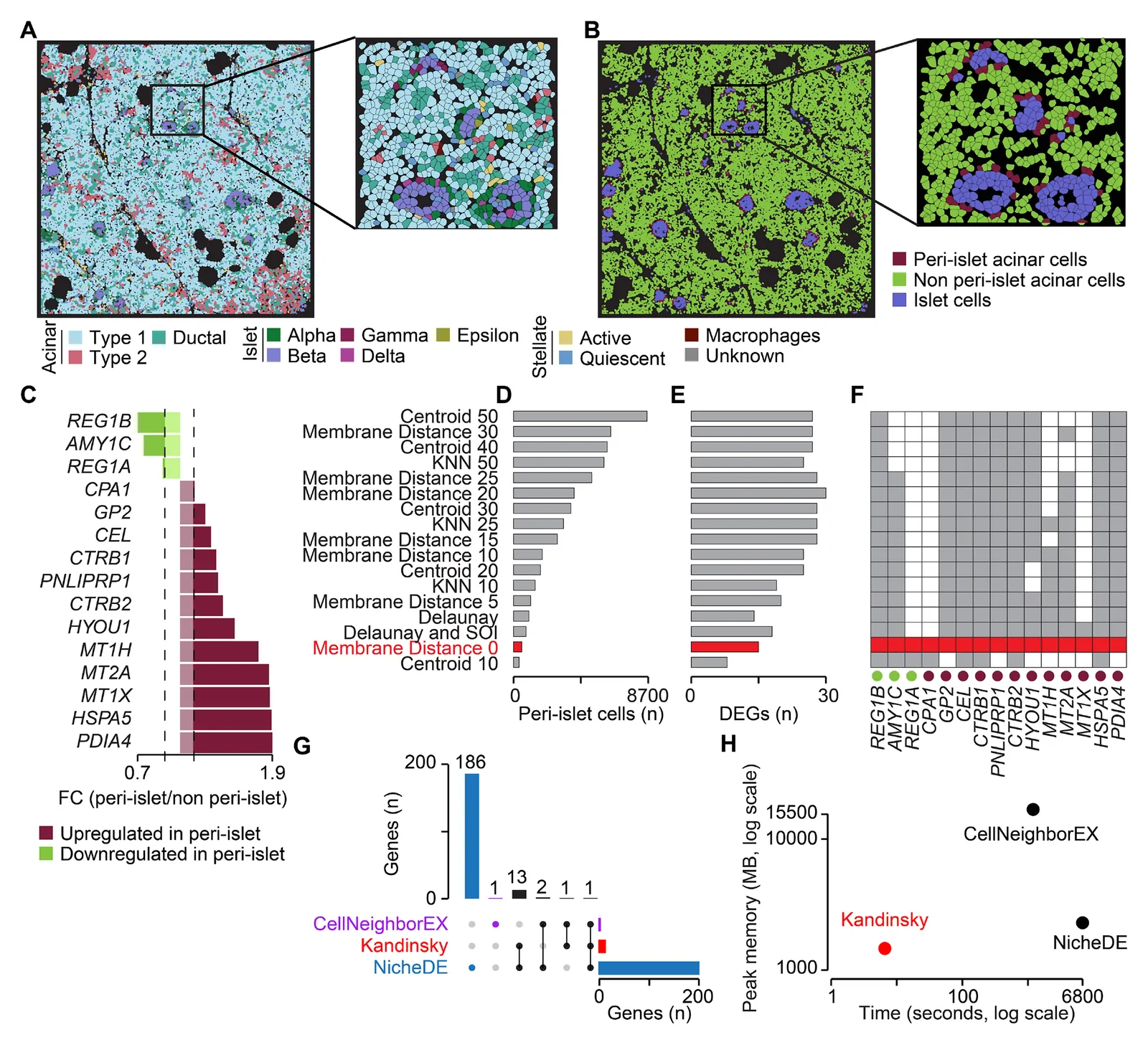
Neighbour-based phenotyping of pancreatic cells. (A, B) Cells from nine representative FOVs of a human pancreas coloured using the original cell type (A) and peri-islet and non peri-islet acinar cell definitions from Kandinsky (B). Inlets show examples of pancreatic islets surrounded by acinar cells; (C) Genes up- (FC > 1.1, FDR < 0.05, Bonferroni correction) and down- (FC < 0.9, FDR < 0.05) regulated in peri-islet acinar cells. DEG analysis was run between peri-islet and non peri-islet acinar cells. Genes expressed in < 20% of peri-islet acinar cells and < 5% of non-peri-islet acinar cells were excluded to limit transcriptional contamination. Number of peri-islet acinar cells (D) and peri-islet DEGs (E) using different c-NB definitions and parameters. C-NB definition used in (C) is in red; (F) Binary matrix showing whether alternative c-NB definitions identified the genes reported in (C) as DEGs (grey, FDR < 0.01); (G) Intersection of peri-islet DEGs across methods; (H) Comparison of computational performance across methods. Median runtime and peak memory values were estimated across 10 iterations. DEG: differentially expressed gene; FC: fold change; FDR: false discovery rate; FOV: field of view; KNN: K-nearest neighbour; MB: megabyte; SOI: sphere of influence.

To test the differential expression module (Figure 1C), we applied Kandinsky to a CosMx single-cell whole-transcriptome dataset composed of 48,951 normal pancreatic cells from a healthy donor^[47]^. Based on the original annotation^[47]^, we identified 12 cell types corresponding to major exocrine (acinar and ductal) and endocrine (islet) pancreatic cells, as well as stellate cells and macrophages (Figure 2A).

Experiments in mice showed that acinar cells proximal to islets upregulate trypsin and other digestive enzymes^[59]^ and undergo acinar-to-beta cell reprogramming^[60]^. We used Kandinsky to test whether acinar cells located within peri-islet neighbourhoods of the human pancreas showed a transcriptional profile compatible with this reprogramming. We identified c-NBs composed of cells in physical contact with each other (membrane distance = 0, Figure 1B). We then clustered acinar cells into two groups: those in physical contact with at least one islet cell (532 peri-islet acinar cells) and those not in contact with islets (33,465 non peri-islet acinar cells, Figure 2B). Comparing the gene expression profiles of these two groups, we found 15 differentially expressed genes (DEGs, Figure 2C). Among these, digestion-associated (*CTRB1/2, CPA1, CEL, PNLIPRP1*), insulin-sensitive (*PDIA4, MT2A* and *MT1H/X*), and pancreatic progenitor (*GP2*) genes were significantly overexpressed in peri-islet acinar cells (Figure 2C). These cells also showed significantly lower expression of acinar markers (*REG1A* and *REG1B*, Figure 2C). This expression profile is compatible with the reprogramming of peri-islet acinar into beta-like cells observed in mice^[60]^.

To test the stability of these results, we repeated the analysis using 16 different c-NB definitions. Despite the variability in the resulting number of peri-islet acinar cells (Figure 2D) and DEGs (Figure 2E), 11 of the 15 original DEGs, including key cell reprogramming markers, were also identified as differentially expressed by at least 12 of the 16 combinations (Figure 2F). We also compared Kandinsky with two independent methods that can perform spatially resolved differential gene expression analysis (CellNeighborEX v1.0.1^[34]^ and NicheDE v0.0.0.9^[33]^, Table S1). All DEGs identified by Kandinsky were also detected by at least one another method (Figure 2G) and 93% of them were confirmed as differentially expressed by NicheDE (*p* < 2.2 × 10^-16^, two-tailed Fisher’s exact test). In terms of computational performance Kandinsky showed the lowest memory usage and elapsed runtime of the three methods (Figure 2H).

### 3.3 Spatial clustering identifies subpopulations of pancreatic cells within spatial niches

**Figure 3:**
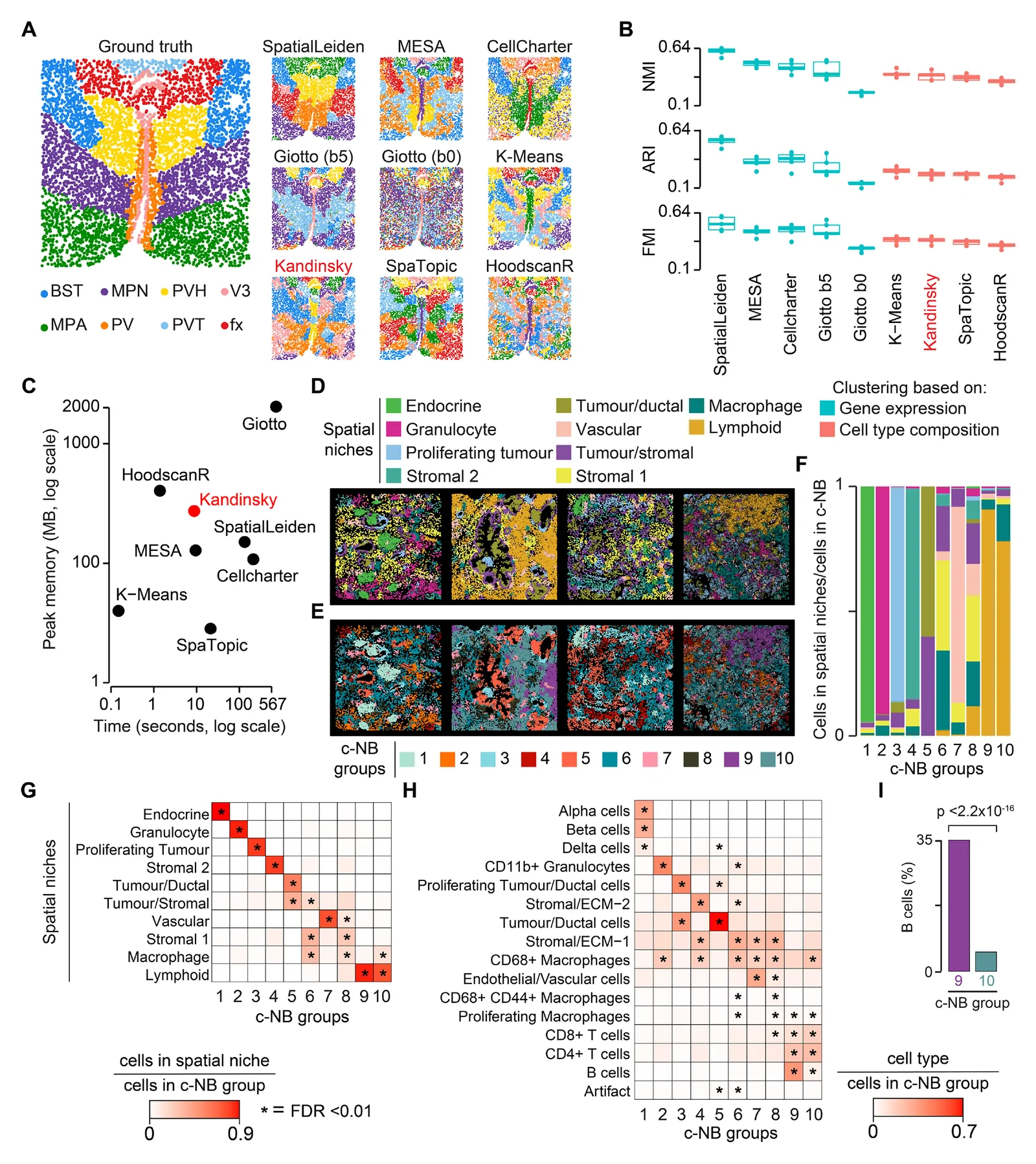
c-NB clustering on MERFISH and IMC datasets. (A) Ground truth annotation and corresponding spatial clusters identified across tools in a representative tissue section (Bregma -0.04)^[4]^; (B) Accuracy in recovering ground truth annotation across methods and tissue sections (*n* = 5); (C) Comparison of computational performance across methods. Median runtime and peak memory values were estimated across 10 iterations; (D, E) Cells from representative FOVs of four PDAC samples (P1-R1, P4-R1, P6-R3, P8-R2) coloured by spatial niches (D) and c-NB clusters (E); (F) Proportion of spatial niches within c-NB clusters; (G, H) Enrichment of spatial niches; (G) and cell types (H) within c-NB clusters. Enrichment was tested using one-tailed Fisher’s exact test. P-values were corrected for multiple testing with the Benjamini-Hochberg method; (I) Comparison of B cell proportions between c-NB clusters 9 and 10. Proportions were compared using two-sided Fisher’s exact test. ARI: adjusted rand index; BST: bed nuclei of the stria terminalis; c-NB: cell neighbourhood; FDR: false discovery rate; FMI: Fowlkes-Mallows index; FOV: field of view; fx: columns of the fornix; IMC: imaging mass cytometry; MB: megabyte; MPA: medial preoptic area; MPN: medial preoptic nucleus; NMI: normalised mutual information; PDAC: pancreatic ductal adenocarcinoma; PV: periventricular hypothalamic nucleus; PVH: paraventricular hypothalamic nucleus; PVT: paraventricular nucleus of the thalamus; V3: third ventricle; MESA: multiomics and ecological spatial analysis.

We tested the ability of the c/s-NBs clustering module (Figure 1C) to reconstruct pre-annotated spatial domains as compared to seven other tools (SpatialLeiden v0.4.0^[40]^, CellCharter v0.3.4^[25]^, MESA v0.1.2^[32]^, Giotto v4.2.3^[24]^, SpatialTopic v1.3.0^[41]^, hoodscanR v.1.8.0^[23]^, and K-means clustering implemented by tools like ISCHIA v1.0.0.0^[26]^, Table S1). As ground truth, we relied on a MERFISH dataset of five thoroughly annotated mouse hypothalamus sections^[4,50]^, for a total of 28,317 single cells and 115 genes (Figure 3A). To assess agreement with the original annotation, we applied three cluster similarity metrics for each tissue section (normalised mutual information (NMI), adjusted Rand index (ARI), and Fowlkes-Mallows index (FMI)). Except for Giotto, clustering approaches based on gene expression showed overall higher concordance with the original annotation than methods based on cell type composition, including Kandinsky (Figure 3B). In terms of computational costs, Kandinsky required intermediate runtime and memory levels (Figure 3C).

Next, we tested whether Kandinsky could detect cell neighbourhoods in an IMC spatial proteomic dataset composed of 144,976 single cells from nine PDAC samples assayed with a panel of 26 metal-tagged antibodies^[52]^. Applying KNN to derive neighbourhoods and K-means for clustering, the original study described ten distinct spatial niches (Figure 3D). To define c-NBs, we first stitched the nine samples, then applied KNN with the same neighbourhood size (*k* = 20) as that of the original study and clustered these into ten groups using the built-in unsupervised c-NB clustering function (Methods). The low number of k-nearest neighbours reduced the possibility of artificial neighbourhood relationships between cells from independent samples. Moreover, we specifically checked that the ROIs were sufficiently distant from each other after stitching.

Overall, we found good concordance between cells assigned to the original spatial niches and newly detected c-NB clusters (adjusted Rand index = 0.4, Figure 3E). Four niches (endocrine, granulocyte, proliferating tumour, and stromal 2) showed significant overlap with only one c-NB group (Figure 3F,G). For the remaining six niches, c-NB clusters refined the original annotation. For instance, cells in the original tumour/ductal and tumour/stromal niches were reassigned to c-NB clusters 5 and 6 (Figure 3G). Enrichment analysis of cell types confirmed that c-NB cluster 5 was mostly composed of tumour cells, while c-NB cluster 6 was mostly composed of a variety of stromal and immune cells (Figure 3H). Similarly, the vascular niche, which in the original study contained also a significant proportion of stromal cells and macrophages^[52]^, was split into c-NB cluster 7 (composed mostly of endothelial cells with a minor contribution of macrophages and stromal cells) and 8 (mostly macrophages and stromal cells with a minor contribution of endothelial cells, Figure 3G,H). Finally, the original lymphoid niche was further split into c-NB clusters 9 and 10 (Figure 3G,H). Both c-NB clusters were enriched in T cells, but c-NB cluster 9 showed a significant overrepresentation of B cells (Figure 3I), while c-NB cluster 10 showed a significant overrepresentation of macrophages (Figure 3G,H). In all these examples, a more precise identification of cell populations within separate groups may improve their functional characterisation.

### 3.4 Myoepithelial cells surround non-invasive cancer cells within the breast tissue

**Figure 4:**
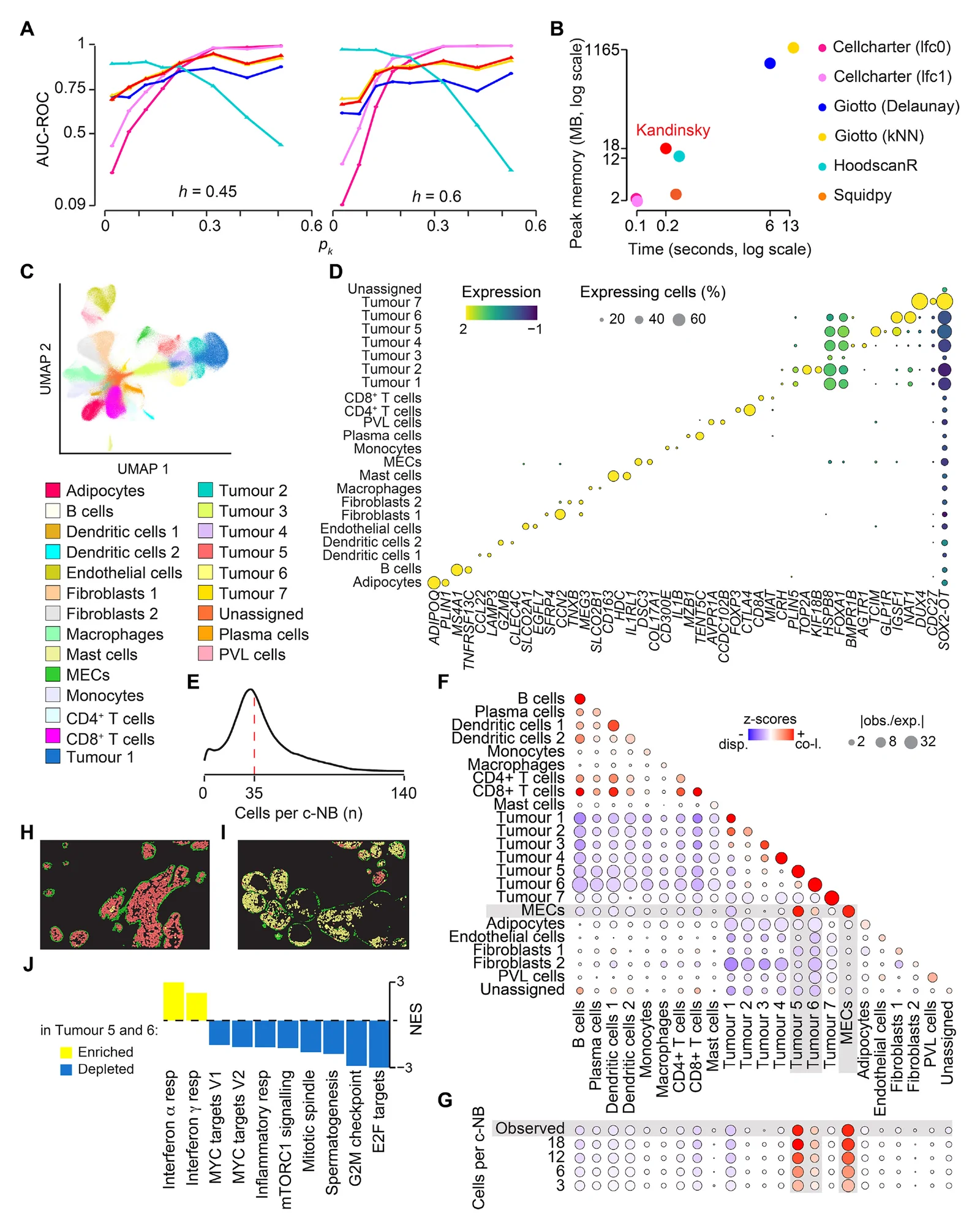
Cell co-localisation in simulated and real breast cancer data. (A) Accuracy across tools in recovering ground truth co-localisation patterns was calculated as AUC-ROC curves for different ct0 abundances (*p*_*k*_) in samples with weak (*h* = 0.45) or strong (*h* = 0.6) ct1-ct0 neighbour preference compared to samples with random (*h* = 0.25) neighbour preference; (B) Comparison of computational performance across methods. Median runtime and peak memory values were estimated across 10 iterations; (C) Unsupervised clustering of 468,583 cells from a breast cancer Xenium 5K dataset^[55]^. Cells are coloured by cell type; (D) Mean expression values of genes representative of cell clusters. Differential gene expression analysis was performed between each cell type and the rest. Up to two differentially expressed genes per cell type (FC > 2, FDR < 0.05) were selected for visualisation; (E) Distribution of cells per c-NB, identified using a centroid distance of 40 μm. Median number of cells is reported as a dashed red line. Multi-class join count z-score matrix assessing co-occurrence or dispersion of each cell type pair (F) and between MECs and the other cell types, considering different numbers of cells per c-NB (G). Positive z-score values indicate co-localisation, negative z-score values indicate dispersion. Dot size indicates the observed-to-expected ratio of join counts. In (G), three independent runs of downsizing were run per condition. Median z-scores and observed-to-expected ratios are reported. Representative regions of the breast tissue sample showing layers of MECs surrounding tumour 5 (H) and tumour 6 (I) cells; (J) Significantly enriched (NES > 0) and depleted (NES < 0) hallmark cancer pathways^[67]^ in cancer cells of tumour 5 and 6 clusters compared to the rest of cancer cells. Enrichment was estimated using ranked GSEA. Pathways with FDR < 0.05 were considered significant. AUC-ROC: area under the receiver operating characteristic curve; c-NB: cell neighbourhood; FC: fold change; FDR: false discovery rate (Benjamini-Hochberg); GSEA: gene set enrichment analysis; MB: megabyte; MECs: myoepithelial cells; NES: normalised enrichment score; PVL: perivascular-like; UMAP: uniform manifold approximation and projection.

We evaluated the cell co-localisation/dispersion module (Figure 1D) against four widely used approaches (hoodscanR v.1.8.0^[23]^, Giotto v4.2.3^[24]^, Squidpy v1.6.6^[27]^, and CellCharter v0.3.4^[25]^, Table S1) using a simulated single cell dataset as ground truth. We quantified the accuracy of each method to assess co-localisation using the area under the receiver operating characteristic curve (AUC-ROC). Kandinsky showed stable accuracy across simulated conditions with variable cell type abundances (*p*_*k*_ from 0.05 to 0.55) and co-localisation strength (*h* = 0.45 and 0.6, Figure 4A). Moreover, it was the second fastest tool, with a memory peak slightly higher than most of the other tools (Figure 4B).

Next, we investigated the spatial cells co-localisation of cells in a stage II-A breast cancer sample profiled with the Xenium Prime 5K Human Panel^[55]^. After removing low-quality data (Methods), we performed unsupervised clustering of the 468,583 remaining cells, leading to the identification of 23 cell populations based on differentially expressed marker genes (Figure 4C,D). We defined c-NBs according to a centroid distance of 40 μm (Figure 1B), resulting in a median of 35 cells per c-NB (Figure 4E). We then quantified the tendency of cells to significantly aggregate or disperse in the tissue using multi-class join count derived z-scores (Figure 1D). As expected, immune cell populations tended to localise proximal to each other and to be separated from both tumour cells and, to a lower extent, the stromal compartment (Figure 4F). Overall, tumour cells also tended to co-localise in the tissue space, except for a small population corresponding to 1.6% of all tumour cells that clustered away from anything else (tumour 7, Figure 4F).

Interestingly, Kandinsky detected spatial co-localisation between myoepithelial cells (MECs) and two tumour clusters (tumour 5 and 6, Figure 4F). We tested the stability of this co-localisation by keeping only a fixed number of cells (3, 6, 12, and 18) per c-NB. Even reducing the number to only three cells per c-NB, MECs still significantly localise with tumour cells in clusters 5 and 6 (Figure 4G). In normal breast, MECs are known to protect the structure and integrity of epithelial cells^[61,62]^. In breast cancer, MECs act as a physical barrier against tumour cell invasion^[63]^, and the structural integrity of the MEC layer is associated with the risk of progression in patients with ductal carcinoma *in situ*^[64,65]^. When inspecting the spatial organisation of MECs in the tissue, we observed that these cells formed a monolayer around duct-like structures formed by tumour 5 (Figure 4H) and tumour 6 (Figure 4I) cells. Compared to the other tumour cells, tumour 5 and 6 cells showed higher interferon response and reduced proliferation potential (Figure 4J). MEC layers surrounding milk ducts are known to gradually be lost during cancer progression because of the increase in cancer cell proliferation and spreading^[66]^. Although direct histological validation is required, it is tempting to speculate that tumour 5 and 6 cells might have a limited invasive potential. This analysis shows how studying the spatial organisation of cell populations in the tissue can inform on functional phenotypes.

### 3.5 CRC areas enriched in lymphocytes and TAMs show high *CD74* expression

We tested the hot/cold area module of Kandinsky (Figure 1E) in a CRC sample profiled with the CosMx 1,000 gene panel^[57]^. Our laboratory recently showed that cytotoxic lymphocytes induce upregulation of interferon genes, including MHC class II invariant chain *CD74*, in TAMs and CRC cells^[57]^. This is clinically relevant because *CD74* is highly expressed in CRC patients who respond to treatment with immune checkpoint inhibitors^[57,68,69]^. We therefore tested whether *CD74* expression levels correlate with the spatial localisation of cancer cells, TAMs, and lymphocytes.

**Figure 5:**
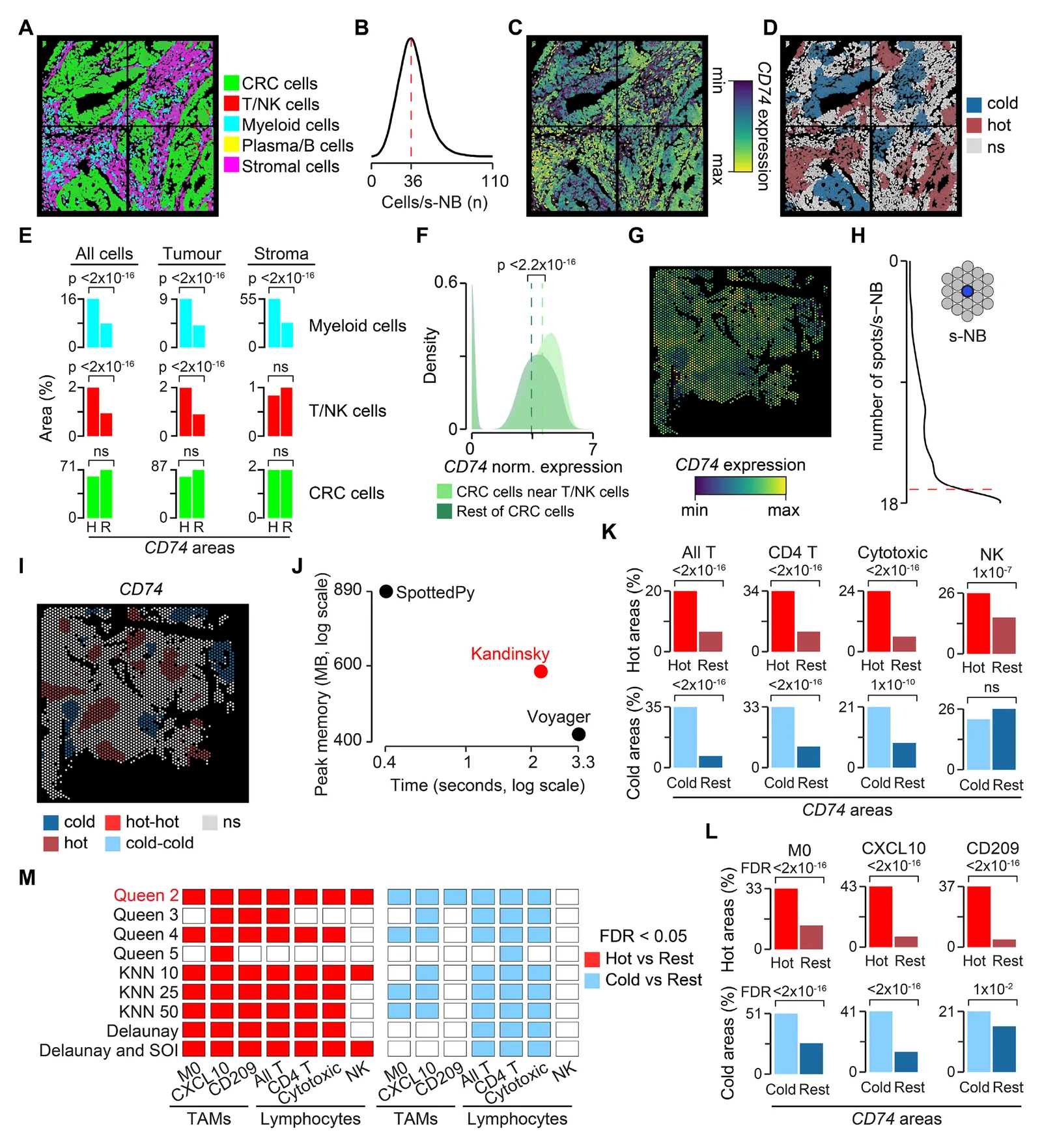
Cell enrichment within *CD74* hot and cold CRC tissue areas. (A) Cells from four representative FOVs of a human CRC sample (CR48) coloured by the original cell type^[57]^; (B) Distribution of cells per c-NB, identified using a cell membrane distance of 30 μm. Median number of cells is reported as a dashed red line; (C, D) Cells from the FOVs shown in (A), coloured by *CD74* expression levels (C) and *CD74* hot or cold areas (D). *CD74* hot and cold areas were identified using the Getis-Ord Gi statistics applied to neighbouring cells; (E) Comparison of *CD74* hot and non-hot areas containing myeloid, T/NK, or CRC cells in the whole CRC sample or considering only tumour and stroma regions; (F) Density plot of *CD74* expression values in CRC cells with or without T/NK cells within their c-NBs. Distributions were compared using two-sided Wilcoxon’s rank-sum test; (G) Visium spots for the CRC sample (CR48), coloured by *CD74* expression levels; (H) Distribution of spots per s-NB, identified using the queen contiguity method with the first two contiguous layers surrounding each spot. Median number of spots is reported as a dashed red line; (I) Visium spots for the CRC sample (CR48), coloured by *CD74* hot or cold areas. *CD74* hot and cold areas were identified based on the Getis-Ord Gi statistics applied to neighbouring spots; (J) Comparison of computational performance across methods. Median runtime and peak memory values were estimated across 10 iterations. Overlap of hot and cold areas for *CD74* and gene signatures associated with TAM (K) and lymphocyte (L) subpopulations compared to the rest of Visium spots; (M) Binary matrix showing whether alternative s-NB definitions replicated the results shown in (K) and (L). s-NB definition used in (I-L) is in red. Significance in (D) and (I) was tested based on permutations, and cells associated with a positive or negative Getis-Ord Gi statistics with FDR < 0.05 were assigned to *CD74* hot or cold areas, respectively (Methods). Proportions in (E), (K), (L) and (M) were compared using one-sided Fisher’s exact test and corrected for multiple testing when needed. c-NB: cell neighbourhood; CRC: colorectal cancer; FDR: false discovery rate (Benjamini-Hochberg); FOV: field of view; KNN: K-nearest neighbour; H: hot; MB: megabyte; NK: natural killer; ns: not significant; R: rest; s-NB: spot neighbourhood; TAMs: tumour-associated macrophages;

Following the original annotation^[57]^, we found five main cell clusters, corresponding to CRC, T/NK, plasma/B, myeloid, and stroma cells (Figure 5A). We then identified c-NBs applying a cell membrane distance of 30 μm, which resulted in a median of 36 cells per c-NB (Figure 5B). Finally, we applied the Getis-Ord Gi statistics (Figure 1E) on single cell *CD74* expression levels (Figure 5C) to map *CD74* hot and cold areas within the sample (Figure 5D).

To test the overlap between the spatial localisation of CRC, myeloid, and T/NK cells and *CD74* expression levels, we measured the relative enrichment of each cell population in *CD74* hot areas using one-tailed Fisher’s exact test. We found a significant enrichment of myeloid and T/NK cells in *CD74* hot areas (Figure 5E), confirming that TAMs are the major source of *CD74* expression in the tumour stroma upon stimulation by interferon-producing lymphocytes^[57,68]^. To test whether the enrichment was confined to specific tissue regions, we derived tissue masks to separate the tumour from the stroma compartment and repeated the analysis in the two regions separately. We observed comparable enrichments of myeloid and T/NK cells in *CD74* hot areas within and outside the tumour compartment, except for T/NK cells in the stroma (Figure 5E).

Overall, CRC cells showed no significant enrichment in *CD74* hot areas (Figure 5E). We reasoned that this may be due to the fact that only CRC cells proximal to lymphocytes would co-localise within *CD74* high expression areas, since only these CRC cells overexpress *CD74* in response to interferon (IFN) stimulation^[57]^. To test this hypothesis, we compared *CD74* expression between CRC cells in c-NBs also containing T/NK cells and those in c-NBs with no T/NK cells. We confirmed that CRC cells proximal to T/NK cells express significantly higher *CD74* levels than the rest of CRC cells (Figure 5F), thus supporting the initial hypothesis.

Being based on 1,000 genes only, the CosMx panel enabled broad separation of the main immune populations (Figure 5A) but prevented further characterisation of relevant immune subpopulations associated with *CD74* hot and cold areas. To overcome this limitation, we profiled another tissue region of the same CRC sample using the Visium whole transcriptomics approach (Methods). After quality control and removal of low quality spots and genes, we mapped *CD74* expression levels across the retained 2,955 spots (Figure 5G). To define s-NBs, we used the queen contiguity approach (Figure 1B) considering the first two contiguous layers surrounding each spot, resulting in a median of 17 spots per s-NB (Figure 5H).

Like the c-NBs analysis, we used s-NBs to identify *CD74* hot and cold areas using the Getis-Ord Gi statistics (Figure 5I). Compared to two other methods that can perform similar analyses (Table S1), Kandinsky showed competitive performances, using less memory than SpottedPy v0.1.1^[30]^ and havinga faster runtime than Voyager v1.8.1^[29]^ (Figure 5J).

Since the Visium technology does not provide single cell resolution, we could not assess the enrichment of TAMs and lymphocytes in *CD74* hot and cold areas directly. Instead, we used seven manually curated gene signatures representing four lymphocyte subpopulations (cytotoxic lymphocytes, T, NK, and CD4 T cells) and three TAM subpopulations (M0, CXCL10, and CD209 TAMs)^[57]^. We identified hot and cold areas in the CRC tissue for each of these gene signatures using the Getis-Ord Gi statistics and applied one-tailed Fisher’s exact test to assess their overlap with *CD74* hot and cold areas. Overall, we found significant enrichments of TAMs (Figure 5K) and lymphocytes (Figure 5L) in hot *CD74* areas, as well as their significant depletions in cold *CD74* areas, except for NK cells. To test the robustness of these associations, we repeated the analysis with eight different s-NB definitions. Except for NK cells in *CD74* hot areas and CD209 TAMs and NK cells in *CD74* cold areas, all other significant associations were supported by at least four other s-NB definitions (Figure 5M). These results confirmed *in situ* the *CD74* overexpression in TAM subpopulations induced by T cells previously detected *in vitro*.

## 4 Discussion

Kandinsky is a computational toolkit designed for cell or spot neighbourhood analysis that allows the user to interact with data derived from several spatial technologies and adapt the analysis to the biological query. Compared to other methods, Kandinsky can identify and use c/s-NBs with greater flexibility. In addition, it implements a suite of functions as well as internal and external analytical modules to facilitate spatial data analysis without depending on multiple software for each specific task.

We showed with real-world data how downstream analytical modules implemented in Kandinsky make use of c/s-NBs in a coherent way, applying c/s-NB definition to each analytical task. Users can interrogate c/s-NBs to define groups of cells and spots proximal to cell types of interest, or c/s-NBs sharing a similar neighbourhood composition, study spatial co-localisation patterns between cell or spot types, or identify local hot or cold expression areas. By analysing spatial datasets generated through CosMx, IMC, Xenium, and Visium platforms, we demonstrated Kandinsky’s versatility to produce biologically meaningful results supported by literature evidence.

One of the challenges in analysing spatial omics data is spatial autocorrelation^[70]^. In Kandinsky, we implemented an approach to control for this based on random c/s-NB downsizing. Future releases may include more complex approaches based on generalised additive models or mixed models to account for both spatial autocorrelation and sample of origin^[71]^. Alternatively, spatially aware data sketching could be used to reduce sample size without affecting tissue representation^[72]^.

Although c/s-NB grouping, co-localisation, and dispersion analyses are compatible with spatial data of any resolution, they do not support direct inclusion of cell type deconvolution often associated with spot-level data. This is a current limitation of Kandinsky that could be part of future updates. Another possible improvement would be the possibility to run Kandinsky on more than one dataset at the same time for multi-sample comparisons. In its current version, Kandinsky can handle independent samples within the same dataset, but it cannot merge samples from independent Seurat objects. This requires a substantial change to the Kandinsky object format to store molecular and spatial information for independent samples, without losing interoperability with the underlying Seurat infrastructure.

Given the continuous development of new spatial technologies, we aim at maintaining Kandinsky code and documentation up to date and expanding the list of compatible spatial platforms and possible analyses.

## Supplementary Material

The supplementary material for this article is available at: Supplementary materials.

Supplementary materials

## Data Availability

CosMx human pancreas data were downloaded from https://nanostring.com/products/cosmx-spatial-molecular-imager/ffpe-dataset/cosmx-smi-human-pancreas-ffpe-dataset. Annotated MERFISH mouse hypothalamus dataset was downloaded from SDMBench website (http://sdmbench.drai.cn/). IMC PDAC data were downloaded from https://zenodo.org/records/10246315. Simulated spatial dataset was downloaded from https://github.com/SchapiroLabor/NEP_comparison/tree/main/simulated_data/. Xenium breast cancer data were downloaded from https://www.10xgenomics.com/datasets/xenium-prime-ffpe-human-breast-cancer. CosMx CRC data were downloaded from https://zenodo.org/records/10927005. Visium CRC data have been deposited in Zenodo (https://doi.org/10.5281/zenodo.15209564). Access to these data is restricted to non-commercial research only and requires data sharing agreement with the Francis Crick Institute. Kandinsky is freely available as an R package at https://github.com/ciccalab/Kandinsky. A link to the repository containing code and output files from the benchmarking is available on the same website. Tutorials reproducing the results described in this study and the manual for Kandinsky functions are available at https://ciccalab.github.io/Kandinsky/index.html.
